# Resveratrol Prevents Dendritic Cell Maturation in Response to Advanced Glycation End Products

**DOI:** 10.1155/2013/574029

**Published:** 2013-07-09

**Authors:** Brigitta Buttari, Elisabetta Profumo, Francesco Facchiano, Elif Inci Ozturk, Luca Segoni, Luciano Saso, Rachele Riganò

**Affiliations:** ^1^Department of Infectious, Parasitic and Immune-Mediated Diseases, Istituto Superiore di Sanità, 299 Viale Regina Elena, 00161 Rome, Italy; ^2^Department of Hematology, Oncology and Molecular Medicine, Istituto Superiore di Sanità, 00161 Rome, Italy; ^3^Department of Pharmacology, Faculty of Pharmacy, Hacettepe University, 06100 Ankara, Turkey; ^4^Department of Physiology and Pharmacology “Vittorio Erspamer”, La Sapienza University of Rome, 00185 Rome, Italy

## Abstract

Advanced glycation end products (AGEs), generated through nonenzymatic glycosylation of proteins, lipids, and nucleic acids, accumulate in the body by age thus being considered as biomarkers of senescence. Senescence is characterized by a breakdown of immunological self-tolerance, resulting in increased reactivity to self-antigens. Previous findings suggest that AGE and its receptor RAGE may be involved in the pathogenesis of autoimmune reactions through dendritic cell (DC) activation. The aim of this study was to investigate whether resveratrol, a polyphenolic antioxidant compound with tolerogenic effects on DCs, was able to counteract the mechanisms triggered by AGE/RAGE interaction on DCs. By immunochemical and cytofluorimetric assays, we demonstrated that *in vitro* pretreatment of human monocyte-derived DCs with resveratrol prevents DC activation in response to glucose-treated albumin (AGE-albumin). We found that resveratrol exerts an inhibitory effect on DC surface maturation marker and RAGE up-regulation in response to AGE-albumin. It also inhibited proinflammatory cytokine expression, allostimulatory ability upregulation, mitogen-activated protein (MAP) kinases, and NF-*κ*B activation in AGE-albumin-stimulated DCs. We suggest that resveratrol, by dismantling AGE/RAGE signaling on DCs may prevent or reduce increased reactivity to self-molecules in aging.

## 1. Introduction

Advanced glycation end products (AGEs) are a heterogenous group of molecules that are generated through nonenzymatic glycosylation (glycation) and oxidation of proteins, lipids, and nucleic acids [1]. Even though glycation is physiologically present and is modulated by several factors, disorders of glucose metabolism and systemic autoimmune diseases associated with inflammation and oxidative stress may favour the formation and accumulation of these products [2–6]. It is noteworthy that AGEs are normally produced in the body and they accumulate by age thus being considered as biomarkers of senescence [7]. Further, AGE adverse effects on cellular and tissue functions arise from their potential to cross-link intracellular and extracellular proteins thus altering their structure and function and triggering the development of different age-associated diseases, such as neurodegenerative and cardiovascular diseases [8, 9]. Proteins modified by glycation have been shown to become antigenic thus inducing activation of immune responses [10–12]. Six receptors that recognize and bind AGEs have been identified [13, 14], among which the best characterized and most extensively studied receptor being RAGE, a 46-kDa protein, mainly expressed on the surface of endothelial cells, smooth muscle cells, and monocyte-derived dendritic cells (DCs) [15, 16]. Although there is accumulating evidence that AGEs are involved in senescence [9], nevertheless further investigations are needed to clarify the role of glycation in aging and aging-related diseases. It is known that the immune system undergoes continuous morphological and functional changes throughout the lifetime and gradually declines with age [17]. The decline in protective immune responses to exogenous and infectious agents is accompanied by an increased reactivity towards self- or endogenous antigens [17]. The mechanisms underlying the breakdown of immunological self-tolerance are not yet fully understood. Dendritic cells, the most potent antigen-presenting cells (APCs), have a pivotal role in the onset and regulation of adaptive immune response and in the induction of autoimmunity [18]. Previous studies demonstrated that AGE-modified serum molecules induced maturation of DCs and augmented their capacity to stimulate T-cell proliferation and cytokine secretion possibly through upregulation of RAGE [11, 12]. These findings suggest that AGE and its receptor (RAGE) axis may be involved in the pathogenesis of age-related autoimmune reactions through DC activation. Disrupting this axis may have a beneficial effect in longevity because many pathological mechanisms triggered by AGE/RAGE interaction can be prevented. Previous studies demonstrated that resveratrol, a natural polyphenol phytoalexin, prevents the AGE-induced acceleration of macrophage lipid accumulation through suppression of RAGE [19] and renders DCs tolerogenic upon activation [20]. Considering this previous evidence, the aim of our study was to investigate whether resveratrol exerts an inhibitory effect on AGE-induced activation of DCs. By immunochemical and flow cytometric analysis we determined the effects of resveratrol on phenotype and functions of human monocyte-derived DCs *in vitro* stimulated with AGE-albumin.

## 2. Materials and Methods

### 2.1. Reagents

A highly purified preparation of bovine serum albumin (Sigma-Aldrich, Milan, Italy) was dissolved in glycation buffer solution (GB) (0.144 g/l KH_2_PO_4_, 0.426 g/l Na_2_HPO_4_) pH 7.4, at 10 *μ*g/mL final concentration, and immediately frozen at −80°C, under sterile conditions. Then albumin aliquots were incubated, under sterile conditions, in the dark, at 37°C for 10, 30, or 60 days, in sealed vials containing either the same concentrations (250 mM) of D-glucose (Sigma-Aldrich) or the nonreducing sugar D-mannitol (Sigma-Aldrich) used as iso-osmotic control, as described [21]. 

Endotoxin contamination in albumin, determined by the quantitative chromogenic limulus amebocyte lysate assay (QCL-1000; BioWhittaker, Walkersville, MD, USA), was less than 0.05 EU/mL of protein. Polymyxin B was added to the cell culture medium at 10 *μ*g/mL, concentration that completely neutralizes the activity of these amounts of lipopolysaccharide (LPS) in all experiments involving albumin. 

Resveratrol was purchased from Sigma. All the chemicals used were of the highest available purity. 

### 2.2. Characterization of AGE-Albumin

#### 2.2.1. SDS-PAGE Analysis

Aliquots (50 *μ*g) of glucose-treated albumin, mannitol-treated albumin, and native albumin were subjected to SDS-PAGE using 8–12% acrylamide gradient gels prepared as described [22]. Nonspecific binding of glycated proteins was minimized by saturation of plastic surfaces and check of the protein recovery after each manipulation. The protein molecular weight standards were provided by Novex (sharp standard molecular weight, Invitrogen, Carlsbad, CA, USA).

#### 2.2.2. AGE Fluorescence Studies

Fluorescence studies were carried out as reported [12]. Briefly, albumin (20 *μ*g/200 *μ*L final volume in GB) was incubated at 37°C in the presence or absence of glucose or mannitol for increasing time in the dark under sterile conditions and constant temperature (21°C), and steady-state fluorescence emission spectra were collected with a FluoroMax-2 spectrofluorometer (Jobin Yvon-Spex, Edison, NJ, USA) using an excitation wavelength of 370 nm and collecting the emission data between 400 and 650 nm at 20°C (equal band widths for excitation and emission 5/5). The emission peak was recorded at 440 nm as reported [23].

#### 2.2.3. Size Exclusion Chromatography

Fast protein liquid chromatography (FPLC) (Pharmacia, Uppsala, Sweden) analysis was carried out to evaluate under native conditions the albumin and AGE-albumin molecular size. After the incubation with sugars, aliquots of 50 *μ*L (1 mg/mL protein concentration) were injected onto a Superose S12 Pharmacia column equilibrated in Phosphate buffer saline without calcium and magnesium PBS^−/−^, pH 7.4. Elution was carried out with a 0.4 mL/min flow rate at room temperature, and protein peaks were detected under UV recording (optical density at 280 nm). The column was calibrated with a mixture of protein molecular weight standards (Pharmacia) according to the manufacturer's instructions.

#### 2.2.4. Bioinformatic Analysis of Albumin Glycation

The potentiality of albumin (Bos taurus, accession number AAA51411), to be glycated and to generate AGE product, was studied by means of a web source (NetGlycate 1.0 server) able to predict glycation of *ε* amino groups of lysines in proteins. Functional sites were analyzed by means of bioinformatic tools [24]. Structural analyses and computer-assisted molecular simulations were carried out as described [25].

### 2.3. Generation of DCs and T Lymphocytes

Blood samples from 5 healthy blood donors from the Transfusion Center at La Sapienza University of Rome were used to obtain peripheral blood mononuclear cells (PBMCs). The study was conducted in accordance with the Helsinki Declaration of 1975 and 1983.

Monocytes and immature dendritic cells (iDCs) were obtained from PBMCs, as previously described [12]. Immature DCs were stimulated with 0.2 *μ*g/mL LPS (strain 0111:B4 *Escherichia coli*, Sigma-Aldrich) for 18 hours to obtain LPS-matured DCs. The purity of iDCs was found higher than 95%, as assessed by flow cytometric analysis (FACSCanto using CellDIVA, BD-Biosciences, San Diego, CA, USA) of cells stained with a mixture of CD14-fluorescein isothiocyanate (FITC) and CD1a-phycoerytrin (PE) monoclonal antibodies (mAbs) (PharMingen, San Diego, CA, USA). CD4^+^ T cells were purified from PBMCs by magnetic selection using the anti-CD4^+^ microbeads (Miltenyi Biotec Belgish, Gladbach, Germany), according to the manufacturer's instructions. The purity of positively selected CD4^+^ T cells was higher than 95%, as assessed by flow cytometric analysis.

### 2.4. Flow Cytometric Analysis of Phenotypic DC Maturation

Preliminary dose-response experiments (0–200 *μ*g/mL) established that AGE-albumin effects on DC phenotypic maturation were dose dependent: we determined 30 *μ*g/mL as the optimal reagent concentration for DC stimulation. Five-day human iDCs were stimulated with AGE-albumin (30 *μ*g/mL), albumin (30 *μ*g/mL), and LPS (0.2 *μ*g/mL) for 18 hours or left unstimulated. AGE-albumin-stimulated DCs had been pretreated or not with resveratrol for 1 hour at 37°C, 5% CO_2_ at concentrations ranging from 3 to 80 *μ*M. After stimulation, DCs were collected, washed and stained with PE-conjugated mAbs to CD1a, CD80, and human leukocyte antigen-D region related (HLA-DR), and FITC-conjugated mAbs to CD83, CD86, and CD40 (PharMingen) and with the mouse anti-human RAGE mAb (Chemicon International, Inc. Temecula, CA, USA) or with isotype-matched control mAb for 30 minutes at 4°C. To assess RAGE surface expression, cells were then washed and stained with FITC-conjugated goat anti-mouse Ab (Sigma-Aldrich) for 30 minutes on ice. All samples were analyzed by flow cytometry on a FACSCanto using CellDIVA software (BD-Biosciences). 

### 2.5. Sodium Dodecyl Sulphate-Polyacrylamide Gel Electrophoresis (SDS-PAGE) and Western Blot

Cell lysates of 1 × 10^6^ unstimulated or stimulated DCs were mixed with loading buffer (Roth, Karlsruhe, Germany), heated for 5 min at 95°C, and subjected to SDS-PAGE on a 10% polyacrylamide gel with 0.1% SDS using standard procedures (constant voltage at 200 V; 50 *μ*g protein/lane). Proteins were blotted onto polyvinylidenfluoride membrane (Millipore, Bedford, MA, USA) using a semidry blotting unit (Trans-Blot SD; Bio-Rad, München, Germany) in a Tris/Glycin buffer. After transfer, the membrane was blocked in blocking buffer (PBS containing 0.1% Tween-20 and 5% non-fat-dry milk powder) for 1 hour. For detection of RAGE, the membrane was incubated with rabbit anti-human RAGE polyclonal Ab (Chemicon International) at a dilution of 1 : 1000 in blocking buffer overnight. Bound antibodies were visualized with horseradish peroxidase- (HRP-) conjugated goat anti-rabbit immunoglobulin G Ab (1 : 5000; BioRad), and immunoreactivity was assessed by the chemiluminescence reaction using the enhanced chemoluminescence (ECL) Western blotting system (Amersham Life Science). Densitometric analysis was performed using an IMAGEJ 1.43 software.

### 2.6. Cytokine Production

Culture supernatants were collected at 18 hours after DC stimulation with AGE-albumin (30 *μ*g/mL), albumin (30 *μ*g/mL), or LPS (0.2 *μ*g/mL). AGE-albumin-treated DCs had been pretreated or not with resveratrol (50 *μ*M). Levels of IL-12p70, TNF-*α*, IL-10, and IL-1*β* were determined by ELISA (OptEIA kits; BD-Biosciences) following the manufacturer's instructions. The limits of detection were as follows: IL-10, TNF-*α*, and IL-1*β*: 16 pg/mL; IL-12p70 : 7.8 pg/mL.

### 2.7. Mixed Lymphocyte Reaction and IFN-*γ* ^+^ CD4^+^ T-Cell Proliferation

Because features of DC function *in vivo* are critical for antigen presentation and T-cell activation, we evaluated the allostimulatory ability of unstimulated or stimulated DCs in a standard mixed lymphocyte reaction (MLR). LPS-stimulated DCs were used as positive controls. Allogeneic T cells (1 × 10^5^ cells/well) were incubated with irradiated DCs (30 Gy) for 3 days at different responder/stimulator ratios (1 : 4 to 1 : 64 DC : T) in a 96-well round bottom plate. On day 2, 0.5 *μ*Ci/well of [^3^H]-methyl-thymidine (Amersham) was added to each well. After additional 18 hours at 37°C, cells were harvested on a glass fiber filter paper (Wallac, EG&G Company, Turku, Finland), using an automatic cell harvester (Harvester 96, MACH III M, TOMTEC Orange, CT, USA). [^3^H]-methyl-thymidine uptake into cell DNA was measured by reading samples in a *β* counter (1450 Microbeta Plus, Wallac). Net counts per minute (cpm) of triplicate cultures were measured. 

To determine proliferating IFN-*γ* ^+^ CD4^+^ T cells, allogenic CD4^+^ T cells were stained with 5-(and 6-) carboxyfluorescein diacetate, succinimidyl ester (CFDA-SE, Invitrogen, Milan, Italy). Briefly, cells were extensively washed and resuspended at final concentration of 10^7^/mL in PBS. CFDA-SE was added at a final concentration of 2.5 *μ*M and incubated for 4 min at room temperature. The reaction was stopped by washing the cells with RPMI 1640, containing 10% heat-inactivated FBS. CFDA-SE-labeled CD4^+^ T cells were incubated with irradiated stimulated and unstimulated DCs for 3 days at 1 : 32 DC : T cell ratio in RPMI medium containing 10% FBS serum. Cells were monitored on day 3 for CFDA-SE content and IFN-*γ* cytokine expression by flow cytometry. In brief, 10^6^ cells were stimulated with 10^−7 ^M phorbol 12-myristate 13-acetate (PMA) plus 1 *μ*g/mL ionomycin for 4 hours in the presence of 10 *μ*g/mL brefeldin A (all reagents from Sigma-Aldrich). Cells were labeled with anti-CD4 peridinin-chlorophyll-protein (PerCP) (BD-Biosciences) (5 *μ*L/10^4^ cells, 30 minutes on ice) and treated with FACS lysing solution and then with FACS permeabilizing solution (BD-Pharmingen Biosciences). Cells were then stained with a predetermined optimal concentration of anti-IFN-*γ* mAb or of the appropriate isotype control mAb (BD-Biosciences) and analyzed on FACSCanto. The percentage of CD4^+^ CFDA-SE^+^ T cells (proliferating cells) on the IFN-*γ* positive cell gate was evaluated. Cells were gated according to their light scatter properties to exclude cell debris. A minimum of 10,000 viable cells was analyzed for each sample.

### 2.8. Mitogen-Activated Protein (MAP) Kinase p38 and ERK Assay

The fast activated cell-based ELISA MAPK assay kits were used to monitor p38 and ERK activation according to manufacturer's recommendation (Active Motive, Rixensart, Belgium). In brief, iDCs were cultured and seeded in 96-well plates at 5 × 10^4^ cells/well. Cells were stimulated for different times (0–60 minutes) with AGE-albumin (30 *μ*g/mL) after pretreatment or not with resveratrol (50 *μ*M), with albumin (5 mM), LPS (0.2 *μ*g/mL), or PMA (0.2 *μ*g/mL). The number of cells in each well was counted and normalized using the crystal violet solution. The results were expressed as arbitrary units.

### 2.9. Nuclear Factor-*κ*B (NF-*κ*B) Translocation

The NF-*κ*B (p65 and p50) transcription factor assay kit (Active Motive Carlsbad, CA, USA) was used to monitor NF-*κ*B activation. Unstimulated DCs and DCs stimulated for 45 min at 37°C, in 5% CO_2_ with AGE-albumin (30 *μ*g/mL), after pretreatment or not with resveratrol (50 *μ*M) and with albumin (30 *μ*g/mL), were lysed. Protein content was quantified, and activated levels of p65 and p50 subunits were determined in equal amounts of lysates by the use of Abs directed against the subunits bound to the oligonucleotide containing the NF-*κ*B consensus binding site. As a positive control we used a HeLa cell extract and NF-*κ*B wild-type and mutated consensus oligonucleotides to monitor the specificity of the assay, according to manufacturer's instructions. 

### 2.10. Statistical Analysis

Mean values and standard deviations were calculated for each variable under study. All the statistical procedures were performed by GraphPad Prism software (San Diego, CA, USA). Data were analyzed with the Kolmogorov-Smirnov test to verify Gaussian distribution. Normally distributed data were analysed using one-way ANOVA with a Bonferroni *post hoc* test to evaluate the statistical significance of intergroup differences in all the tested variables. *P* values < 0.05 were considered statistically significant.

## 3. Results

### 3.1. Characterization of Glucose-Treated Albumin

**Figure 1:**
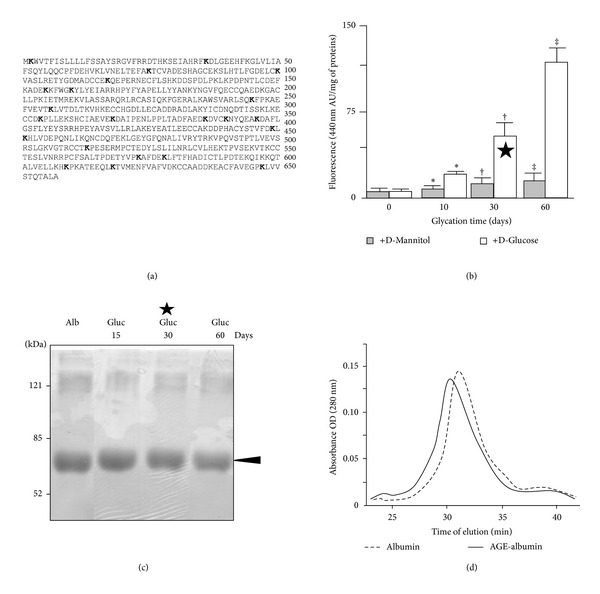
Molecular characterization of glycated albumin. (a) Structural analysis of the amino acid sequence of albumin. The analysis indicates that 22 (in bold) out of 60 lysine residues (K) are potential glycation sites. (b) Fluorescent AGE formation in an albumin solution incubated in the presence of D-glucose or D-mannitol (250 mM) after 10, 30, or 60 days. Data are expressed as means of arbitrary unit/mg of proteins ± SD (*n* = 3). *P < 0.05; ^†‡^ *P* < 0.001. The black star indicates the moderately modified AGE-albumin used in the subsequent cellular studies. (c) SDS-PAGE analysis of albumin preparations (50 *μ*g per lane) incubated or not with 250 mM glucose for the reported times, followed by Coomassie staining, according to standard protocol. (d) Size exclusion chromatography of albumin and AGE-albumin. Albumin was incubated for 30 days with 250 mM D-mannitol (albumin) or D-glucose (AGE-albumin). One representative experiment out of three is reported.

Preliminary evaluation of potential glycation sites within serum albumin sequence was assessed through a bioinformatic analysis showing that 22 out of 60 lysine residues have a significant probability to be nonenzymatically glycosylated (Figure 1(a)). Serum albumin was incubated for increasing time in the presence of D-mannitol or D-glucose as described in Material and Method section. In order to achieve a preliminary molecular characterization of the AGE-albumin preparations, fluorescence, denaturing SDS-PAGE, and native size exclusion chromatographic analyses were performed (Figure 1). The fluorescence at 440 nm, specific for AGE formation, was measured and confirmed the creation of time-dependent AGE products (Figure 1(b)). Of note, doubling the time of sugar exposure (60 *versus* 30 days) induced a 2-fold increase of fluorescence, as expected for a nonsaturated reaction. This result also indicated that the grade of sugar-induced albumin modification at 30-day exposure was not very high (moderately AGE-modified albumin). SDS-PAGE experiments showed only a slight band shift corresponding to a possible 1-2 kDa molecular size increase, likely due to some adducts formation, involving only a few lysine groups (Figure 1(c)). In order to verify whether the AGE-albumin might undergo a significant molecular size modification, a size exclusion chromatographic separation was carried out as described in Section 2, showing the formation of a molecular size increase likely due to some adduct formation (Figure 1(d)). The left shift of the monomeric peak of AGE-albumin sample (Figure 1(d)) has been determined to correspond to about the 11% of protein modification, confirming the low grade of protein AGE modification.

In all the experiments reporting DC maturation and pathways analyses the moderately AGE-modified albumin preparation (30 days with 250 mM glucose, marked by the black star in Figure 1) was used.

### 3.2. Resveratrol Prevents Phenotypical DC Maturation and RAGE Upregulation on Cell Surface in Response to AGE-Albumin

**Figure 2:**
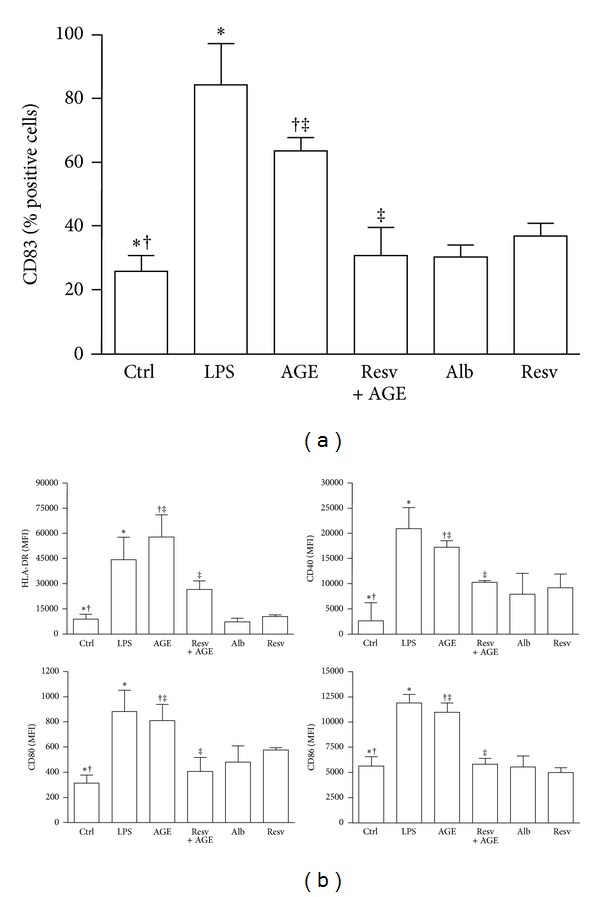
Flow cytometric analysis of phenotypic dendritic cell (DC) maturation. Five-day human DCs pretreated or not with resveratrol (Resv, 50 *μ*M) were cultured for 18 hours with or without AGE-albumin (AGE, 30 *μ*g/mL). DCs treated with LPS (0.2 *μ*g/mL), albumin (Alb; 30 *μ*g/mL), and resveratrol (Resv; 50 *μ*M) were used as controls. Expression of surface molecules was analyzed by flow cytometry as described in Section 2. Phenotypic maturation of DCs was detected by the appearance of CD83 (a) and by the expression of surface molecules (b). After 18 hours of incubation LPS and AGE-albumin induced almost similar DC maturation. Pretreatment of iDCs with resveratrol prevented the phenotypic maturation of DCs induced by AGE-albumin (CD83 and HLA-DR: ^∗†‡^ *P* < 0.001; CD40 and CD80: ^∗†^ *P* < 0.001, ^‡^ *P* < 0.01; CD86: *P < 0.001, ^†‡^ *P* < 0.01). Results are expressed as positive cell percentages (a) and mean fluorescence intensity (MFI, B) (means ± SD, *n* = 4). Samples were analyzed on a FACSCanto cytofluorimeter using CellDIVA (BD-Biosciences). *P* values by one-way ANOVA with a Bonferroni *post hoc* test.

**Figure 3:**
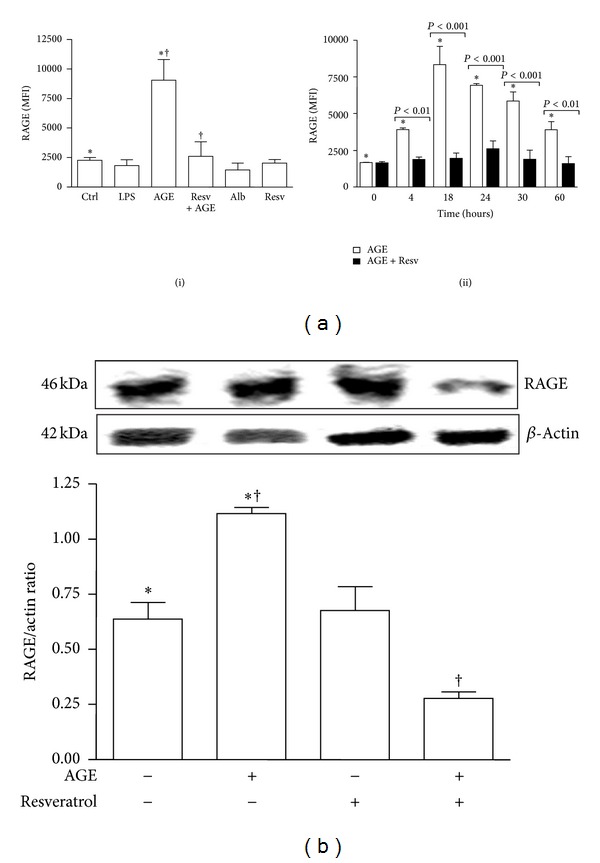
Analysis of RAGE expression on dendritic cells (DCs). Five-day human DCs pretreated or not with resveratrol (Resv, 50 *μ*M) were cultured for 18 hours with or without AGE-albumin (AGE, 30 *μ*g/mL). DCs treated with LPS (0.2 *μ*g/mL), albumin (Alb; 30 *μ*g/mL) and resveratrol (Resv; 50 *μ*M) were used as controls. (a) Flow cytometric analysis of RAGE expression. AGE-albumin (AGE) induced a statistically significant upregulation of RAGE (panel (i)) whose expression remained elevated until 60 hours (panel (ii)). Pretreatment of iDCs with resveratrol prevented RAGE upregulation in response to AGE-albumin at all-time points investigated (panel (ii)). Results are expressed as mean fluorescence intensity (MFI) (means ± SD, *n* = 3). *P < 0.001. ^†^ *P* < 0.05. (b) Western blotting analysis of RAGE expression on DCs. Western blotting followed by densitometric analysis confirmed RAGE downregulation on AGE-albumin-stimulated DCs by resveratrol (means ± SD, *n* = 3). ^∗†^ *P* < 0.001.

Unstimulated DCs showed an immature phenotype (HLA-DR^low^ and CD83^−^) and were weakly immunoreactive for CD80, CD86, CD40, and RAGE. As expected, after 18 hours of incubation, LPS caused DCs to mature, so that CD83 appeared (Figure 2(a)) and HLA-DR, CD80, CD86, and CD40 expression increased (Figure 2(b)). RAGE expression on DC surface remained unchanged (Figure 3(a)). Similarly to LPS, AGE-albumin, but not albumin, induced DC maturation (CD83 and HLA-DR: *P* < 0.001; CD40, CD80, and CD86: *P* < 0.01; Figures 2(a) and 2(b)). AGE-albumin induced also a statistically significant upregulation of RAGE (*P* < 0.001; Figure 3). Of note, RAGE expression on AGE-albumin-stimulated DCs remained elevated until 60 hours (*P* < 0.01; Figure 3(a), panel (ii)). Resveratrol concentration of 50 *μ*M was chosen on the basis of dose-response experiments as the optimal one to modulate DC phenotypic surface marker without affecting cell viability (data not shown). Pretreatment of iDC with resveratrol prevented the appearance of CD83 (*P* < 0.001; Figure 2(a)) and the upregulation of HLA-DR, CD40, CD86, and CD80 (*P* < 0.01, Figure 2(b)). This pretreatment also prevented the upregulation of RAGE after stimulation with AGE-albumin (Figure 3). Western blotting followed by densitometric analysis confirmed cytofluorimetric results (AGE-albumin *versus* AGE-albumin + resveratrol: *P* < 0.001; Figure 3(b)). Interestingly, resveratrol treatment did not affect cell viability, as assessed by trypan blue staining (data not shown).

### 3.3. Resveratrol Prevents Upregulation of Cytokine Production by DCs Stimulated with AGE-Albumin

**Figure 4:**
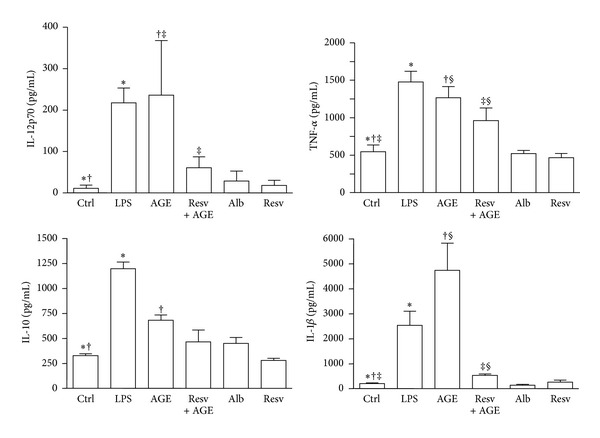
Cytokine production in dendritic cell (DC) culture supernatants. Five-day human DCs pretreated or not with resveratrol (Resv, 50 *μ*M) were cultured for 18 hours with or without AGE-albumin (AGE, 30 *μ*g/mL). DCs treated with LPS (0.2 *μ*g/mL), albumin (Alb; 30 *μ*g/mL), and resveratrol (Resv; 50 *μ*M) were used as controls. Supernatants were collected after 18 hours to measure IL-12p70, TNF-*α*, IL-10, and IL-1*β* by specific ELISA experiments. LPS and AGE-albumin (AGE) triggered a statistically significant upregulation of all cytokine secretions. Pretreatment of iDC with resveratrol prevented the upregulation of all proinflammatory cytokines in response to AGE-albumin. Results are expressed as means ± SD of four independent experiments (IL-12p70: *P < 0.01, ^†‡^ *P* < 0.001; TNF-*α* and IL-1*β*: ^∗†‡^ *P* < 0.001, ^§^ *P* < 0.05; IL-10: ^∗†^ *P* < 0.001).

After 18 hours of culture, AGE-albumin, similarly to LPS, triggered a statistically significant upregulation of IL-12p70, TNF-*α*, IL-10, and IL-1*β* expression (*P* < 0.001; Figure 4). Pretreatment of iDCs with resveratrol (50 *μ*M) prevented the upregulation of all proinflammatory cytokines in response to AGE-albumin (IL-12: *P* < 0.001; TNF-*α* and IL-1*β*: *P* < 0.05; Figure 4), whereas it left IL-10 production expression unchanged. Control albumin and resveratrol alone left cytokine expression unmodified.

### 3.4. Resveratrol Prevents the Allostimulatory Function of DCs Stimulated with AGE-Albumin

**Figure 5:**
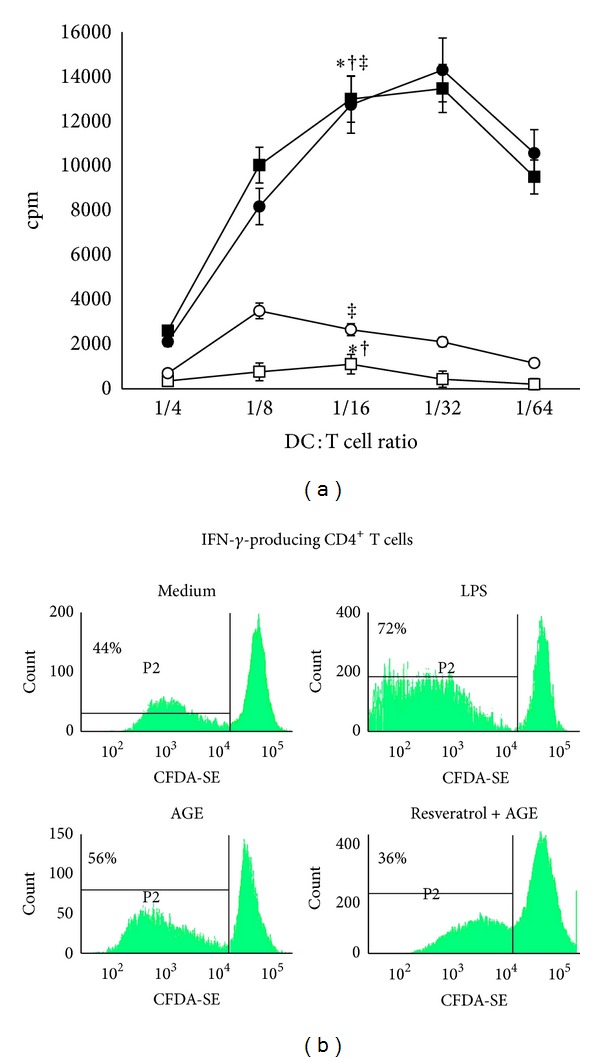
Allostimulatory ability of dendritic cells (DCs). Five-day human DCs were stimulated for 18 hours with LPS (0.2 *μ*g/mL) (■), and AGE-albumin (AGE; 30 *μ*g/mL) (*⚫*), AGE-albumin plus resveratrol (Resv, 50 *μ*M) (◯) or left unstimulated (Ctrl) (□). After 18 hours, DCs were extensively washed and cultured with allogeneic T lymphocytes (1 × 10^5^ cells/well) for 3 days at various stimulator-responder ratios (1 : 4 to 1 : 64 DC/T). (a) Proliferation of allogeneic T cells was measured by [^3^H]-methyl-thymidine incorporation. Data are presented as mean cpm ± SD of four independent experiments (at 1/16 DC/T cells ratio: AGE-albumin and LPS versus unstimulated: ^∗†^ *P* < 0.001; AGE-albumin + resveratrol *versus* AGE-albumin: ^‡^ *P* < 0.001). *P* values by the one-way ANOVA with a Bonferroni *post hoc* test. (b) Proliferation of IFN-*γ*-producing CD4^+^ T cells was determined by staining allogeneic CD4^+^ T cells with CFDA-SE (2.5 *μ*M) and culturing them with irradiated AGE-albumin-stimulated or unstimulated DCs at 1 : 32 DC : T cell ratio. CD4^+^ T-cell proliferative activity (CFDA-SE content), as well as their ability to produce IFN-*γ* was measured on day 3 by flow cytometry as described in Section 2. Figure shows a representative experiment out of 3 with similar results. The numbers show the percentage of proliferating IFN-*γ*-producing CD4^+^ T cells. Samples were analyzed on a FACSCanto cytofluorimeter using CellDiva software (BD-Biosciences).

When irradiated DCs, prestimulated with AGE-albumin, were tested in MLR, the relatively low proliferative ability (mean cpm) of resting allogenic T cells achievable with unstimulated DCs significantly increased, starting from a DC/T cell ratio of 1 : 4 (DC/T cell ratio of 1 : 16: unstimulated versus AGE-albumin-stimulated: *P* < 0.001; Figure 5(a)). This result is similar to that obtained in response to LPS. We observed that pretreatment with resveratrol significantly impaired the allostimulatory function of DCs stimulated with AGE-albumin (at 1 : 16 DC/T cells ratio: *P* < 0.001). Resveratrol alone, when added *in vitro* during DC maturation, did not alter the degree of alloantigen-induced T-cell proliferation observed in response to iDCs (data not shown). 

As a function of T-cell activation, we also measured the percentage of proliferating IFN-*γ*-producing CD4^+^ T cells. Most of IFN-*γ* ^+^ CD4^+^ T cells primed by LPS-matured DCs were proliferating cells (72%) (Figure 5(b)). A high percentage of proliferating IFN-*γ*-producing CD4^+^ T cells was detected also in response to AGE-albumin-matured DCs (56%). In contrast, when CD4^+^ T cells were cultured with resveratrol-pretreated DCs matured in the presence of AGE-albumin, the percentage of proliferating IFN-*γ* ^+^ CD4^+^ T cells resulted lower (56%* versus *36%). 

### 3.5. Resveratrol Prevents MAPK and NF-*κ*B Activation in Response to AGE-Albumin

**Figure 6:**
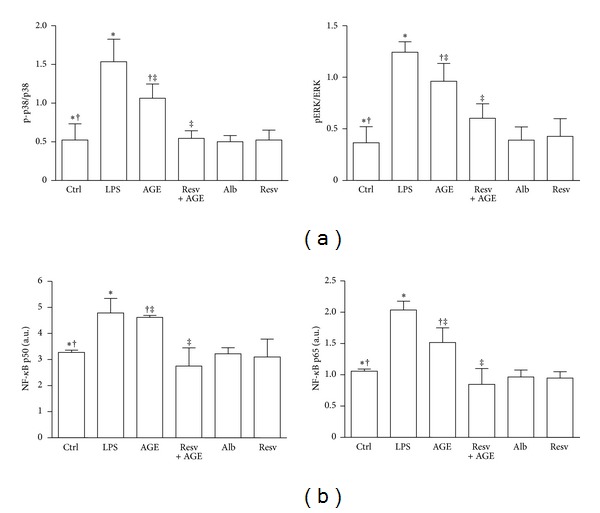
MAPK and NF-*κ*B activation in dendritic cells (DCs). Five-day human DCs pretreated or not with resveratrol (Resv, 50 *μ*M) were cultured for 45 minutes with or without AGE-albumin (AGE, 30 *μ*g/mL). DCs treated with albumin (Alb; 30 *μ*g/mL), resveratrol (Resv; 50 *μ*M), LPS (0.2 *μ*g/mL), or PMA (0.2 *μ*g/mL) were used as controls. Cells were then analyzed by cell-based ELISA MAPK assay to monitor p38 and ERK activation and by NF-*κ*B (p65 and p50) transcription factor assay to monitor NF-*κ*B activation. (a) AGE-albumin stimulation induced the activation of both MAPK p38 and ERK pathways in DCs. Pretreatment of DCs with resveratrol prevented the upregulation of both MAPKs in response to AGE-albumin (*n* = 4; p-p38/p38: ^∗‡^ *P* < 0.001, ^†^ *P* < 0.01; pERK/ERK: ^∗‡^ *P* < 0.001, ^†^ *P* < 0.05). (b) AGE-albumin stimulation significantly increased active p65 and p50 levels in DCs. Pretreatment of DCs with resveratrol prevented the upregulation of both active p50 and p65 in response to AGE-albumin. The results are expressed as arbitrary units (*n* = 4, p50 and p65: *P < 0.05; ^†^ *P* < 0.001).

Increased phosphorylation of p38 and ERK, which peaked at 45 minutes, was observed in AGE-albumin-stimulated DCs in comparison to the unstimulated ones (*n* = 4, p38: *P* < 0.01 and ERK: *P* < 0.05; Figure 6(a)). Pretreatment of iDC with resveratrol prevented the upregulation of both MAPKs in response to AGE-albumin (*P* < 0.001; Figure 6(a)). 

In AGE-albumin-stimulated DCs, active p65 and p50 levels significantly increased in comparison to iDCs (*n* = 4, *P* < 0.05; Figure 6(b)). Pretreatment of iDCs with resveratrol prevented the upregulation of active p50 and p65 in response to AGE-albumin (*P* < 0.001). 

## 4. Discussion

In this study we demonstrated that resveratrol exerts *in vitro* an inhibitory effect on the maturation of human monocyte-derived DCs in response to AGE-albumin by a mechanism that involves reduction of RAGE surface expression, proinflammatory cytokine production, and NF-*κ*B signaling. To the best of our knowledge, this is the first evidence that resveratrol affects DC full maturation in response to an AGE product thus leading to an immature/semimature DC phenotype. 

Resveratrol, a polyphenolic compound found in red wine and grapes, plays a potentially important role in many disorders [26]. It possesses antioxidant, anti-inflammatory, anti-proliferative, and antiangiogenic effects, and many signaling pathways are among its molecular targets. Resveratrol is also believed to be beneficial in increasing the lifespan and healthy aging [26]. Aging is characterized by an erosion of tolerance, resulting in increased reactivity to self-antigens [27, 28]. The knowledge on the underlying mechanisms of resveratrol action on aging, particularly on immunosenescence and autoimmunity, remains incomplete. 

DCs are the most potent APCs and have a pivotal role in the onset and regulation of adaptive immune response. They control Th1/Th2 and Th17/Treg polarization and the state of tolerance to self-antigens. Immature DCs induce regulatory T cells, thus promoting tolerance, whereas mature DCs stimulate effector T cells that support immunity [29–32].

DCs also have the ability to regulate inflammatory responses by secreting cytokines and chemokines [30, 31].

A previous study demonstrated that AGE-albumin induced maturation of DCs and augmented their capacity to stimulate T-cell proliferation and cytokine secretion possibly through upregulation of RAGE and scavenger receptor A [11]. On the basis of this previous evidence we designed the present study to examine the attenuating effects of *in vitro *resveratrol on AGE-albumin-matured DCs. Our bioinformatic analyses of the albumin primary structure indicated that several potential glycation sites are present within the molecule. We verified the occurrence of glycation in glucose-treated albumin by the increase of albumin's molecular dimension and by the formation of a fluorescent AGE. Our preliminary AGE-albumin molecular characterization also indicated that the preparation used in our *in vitro* study was a moderately AGE-modified albumin and that the protein folding modifications likely involved new antigenic features due to the AGE adducts. When we analyzed the phenotypic characteristics of DCs after stimulation with AGE-albumin, we confirmed previous findings on the ability of AGE-albumin to induce DC maturation. Our experiments also demonstrated that the pretreatment of iDCs with resveratrol prevented a complete phenotypic and functional DC maturation in response to AGE-albumin and induced a typical immature/semimature DC phenotype. This phenotype is characterized by a reduced CD83, HLA-DR, CD40, CD80, and CD86 expression associated with low IL-12, TNF-*α*, and IL-1*β* production. In line with their semimature phenotype, AGE-albumin-stimulated DCs pretreated with resveratrol did not increase the low proliferative ability of resting allogenic T lymphocytes in a standard mixed lymphocyte reaction. Further information about the resveratrol inhibitory activity on DCs comes from our experiments investigating RAGE expression on DC surface in response to AGE-albumin. Our results demonstrated that RAGE, the specific receptor for AGEs [13–15], remained down-regulated in AGE-albumin-stimulated iDCs pretreated with resveratrol for more than 48 hours. This last finding could explain how resveratrol may impair maturation of DCs in response to AGE-albumin. It is known that the binding of AGEs to RAGE activates multiple signaling cascades, including Erk1/2 MAPKs, and the generation of reactive oxygen species [33]. These cellular signals may induce activation of downstream effectors such as NF-*κ*B [33]. Under our experimental conditions, resveratrol interferes with RAGE signaling cascade activated by AGE-albumin on DCs through silencing both MAPK p38 and ERK pathways and NF-*κ*B translocation thus leading to the observed immature/semimature phenotype. These findings are in line with the downregulation of MAPK cascade observed on vascular smooth muscle cells [34]. Our results on the inhibitory effect of resveratrol on the NF-*κ*B pathway support previous findings on LPS-matured DCs [20].

Our *in vitro* findings help to explain why self-proteins become immunogenic *in vivo*. 

Nowadays, it is generally agreed that some autoimmune diseases are associated with abnormal presentation of cryptic or neoepitopes of self-antigens by DCs. Because epitope dominance is influenced by protein structure, glycation and glyco-oxidation events may change the molecular context of protein epitopes (for altered secondary or tertiary structure), thus permitting the efficient presentation of cryptic and neodeterminants. This is supported by our observations in which we reported that oxidatively modified proteins increase their immunogenicity [12, 35–39]. Although enhanced in diabetes, AGEs accumulation also occurs in euglycemia, aging [40], and systemic autoimmune diseases [41, 42], albeit to lower degrees, driven by oxidative stress and inflammation or simply by diet-induced postprandial hyperglycaemic peaks. Increased oxidative stress and AGE accumulation may result in the overexpression of RAGE [43, 44]. RAGE expression is increased in an inflammatory milieu and present in aging subjects, who in turn may particularly be exposed to the deleterious effect of AGEs. The AGE-RAGE interaction might act as a proinflammatory loop in these subjects, thus contributing to a chronic low-grade inflammation which is a precursor of aging-related diseases [43].

## 5. Conclusions

Our *in vitro* findings may help to explain the detrimental effects of AGE accumulation during aging, particularly the increased reactivity towards self- or endogenous antigens observed in aged individuals. A possibility is that chronic oxidative stress conditions in aged individuals cause AGE accumulation in the body. The generation of AGEs and augmentation of proinflammatory mechanisms provide a powerful feedback loop for sustained oxidative stress, ongoing generation of AGEs, and autoimmunity. Increased AGE-associated modifications in existing self-molecules may in fact enhance their immunogenic potential and may initiate a local autoimmune process in aged subjects with consequent development of different age-associated diseases. 

Our *in vitro* findings now call for studies in aged individuals to verify the pathogenetic role of glycated proteins, as trigger of specific humoral and cellular immune reactions.

Our results suggest that an antioxidant therapy or a prevented diet with resveratrol, besides inhibiting glycation and glyco-oxidation reactions, may also directly act by dismantling AGE/RAGE signaling, thus preventing or reducing increased reactivity to self-molecules in aging.

## References

[1] Thorpe SR, Baynes JW (2003). Maillard reaction products in tissue proteins: new products and new perspectives. *Amino Acids*.

[2] Ramasamy R, Shi FY, Schmidt AM (2009). RAGE: therapeutic target and biomarker of the inflammatory response—the evidence mounts. *Journal of Leukocyte Biology*.

[3] Shanmugam N, Kim YS, Lanting L, Natarajan R (2003). Regulation of cyclooxygenase-2 expression in monocytes by ligation of the receptor for advanced glycation end products. *The Journal of Biological Chemistry*.

[4] Bierhaus A, Hofmann MA, Ziegler R, Nawroth PP (1998). AGEs and their interaction with AGE-receptors in vascular disease and diabetes mellitus. I. The AGE concept. *Cardiovascular Research*.

[5] Basta G, Schmidt AM, de Caterina R (2004). Advanced glycation end products and vascular inflammation: implications for accelerated atherosclerosis in diabetes. *Cardiovascular Research*.

[6] Smit AJ, Lutgers HL (2004). The clinical relevance of advanced glycation endproducts (AGE) and recent developments in pharmaceutics to reduce AGE accumulation. *Current Medicinal Chemistry*.

[7] Fleming TH, Humpert PM, Nawroth PP, Bierhaus A (2011). Reactive metabolites and AGE/RAGE-mediated cellular dysfunction affect the aging process—a mini-review. *Gerontology*.

[8] Hegab Z, Gibbons S, Neyses L, Mamas MA (2012). Role of advanced glycation end products in cardiovascular disease. *World Journal of Cardiology*.

[9] Baraibar MA, Liu L, Ahmed EK, Friguet B (2012). Protein oxidative damage at the crossroads of cellular senescence, aging, and age-related diseases. *Oxidative Medicine and Cellular Longevity*.

[10] Kurien BT, Scofield RH (2008). Autoimmunity and oxidatively modified autoantigens. *Autoimmunity Reviews*.

[11] Ge J, Jia Q, Liang C (2005). Advanced glycosylation end products might promote atherosclerosis through inducing the immune maturation of dendritic cells. *Arteriosclerosis, Thrombosis, and Vascular Biology*.

[12] Buttari B, Profumo E, Capozzi A (2011). Advanced glycation end products of human *β*2 glycoprotein I modulate the maturation and function of DCs. *Blood*.

[13] Li YM, Mitsuhashi T, Wojciechowicz D (1996). Molecular identity and cellular distribution of advanced glycation endproduct receptors: relationship of p60 to OST-48 and p90 to 80K-H membrane proteins. *Proceedings of the National Academy of Sciences of the United States of America*.

[14] Miyazaki A, Nakayama H, Horiuchi S (2002). Scavenger receptors that recognize advanced glycation end products. *Trends in Cardiovascular Medicine*.

[15] Brett J, Schmidt AM, Yan SD (1993). Survey of the distribution of a newly characterized receptor for advanced glycation end products in tissues. *The American Journal of Pathology*.

[16] Rojas A, Delgado-Lopez F, Gonzalez I, Perez-Castro R, Romero J, Rojas I (2013). The receptor for advanced glycation end-products: a complex signaling scenario for a promiscuous receptor. *Cellular Signalling*.

[17] Agrawal A, Sridharan A, Prakash S, Agrawal H (2012). Dendritic cells and aging: consequences for autoimmunity. *Expert Review of Clinical Immunology*.

[18] Jensen SS, Gad M (2010). Differential induction of inflammatory cytokines by dendritic cells treated with novel TLR-agonist and cytokine based cocktails: targeting dendritic cells in autoimmunity. *Journal of Inflammation*.

[19] Zhang Y, Luo Z, Ma L, Xu Q, Yang Q, Si L (2010). Resveratrol prevents the impairment of advanced glycosylation end products (AGE) on macrophage lipid homeostasis by suppressing the receptor for AGE via peroxisome proliferator-activated receptor *γ* activation. *International Journal of Molecular Medicine*.

[20] Švajger U, Obermajer N, Jeras M (2010). Dendritic cells treated with resveratrol during differentiation from monocytes gain substantial tolerogenic properties upon activation. *Immunology*.

[21] Facchiano F, Lentini A, Fogliano V (2002). Sugar-induced modification of fibroblast growth factor 2 reduces its angiogenic activity in vivo. *The American Journal of Pathology*.

[22] Facchiano F, D’Arcangelo D, Russo K (2006). Glycated fibroblast growth factor-2 is quickly produced in vitro upon low-millimolar glucose treatment and detected in vivo in diabetic mice. *Molecular Endocrinology*.

[23] Wu JT, Tu MC, Zhung P (1996). Advanced glycation end product (age): characterization of the products from the reaction between d-glucose and serum albumin. *Journal of Clinical Laboratory Analysis*.

[24] Facchiano AM, Facchiano A, Facchiano F (2003). Active Sequences Collection (ASC) database: a new tool to assign functions to protein sequences. *Nucleic Acids Research*.

[25] Aguzzi MS, Facchiano F, Ribatti D (2004). A novel RGDS-analog inhibits angiogenesis in vitro and in vivo. *Biochemical and Biophysical Research Communications*.

[26] Catalgol B, Batirel S, Taga Y, Ozer NK (2012). Resveratrol: French paradox revisited. *Frontiers in Pharmacology*.

[27] Agrawal A, Tay J, Ton S, Agrawal S, Gupta S (2009). Increased reactivity of dendritic cells from aged subjects to self-antigen, the human DNA. *Journal of Immunology*.

[28] Agrawal A, Tay J, Yang G-E, Agrawal S, Gupta S (2010). Age-associated epigenetic modifications in human DNA increase its immunogenicity. *Aging*.

[29] Agrawal S, Agrawal A, Doughty B (2003). Cutting edge: different toll-like receptor agonists instruct dendritic cells to induce distinct Th responses via differential modulation of extracellular signal-regulated kinase-mitogen-activated protein kinase and c-Fos. *Journal of Immunology*.

[30] Iwasaki A, Medzhitov R (2010). Regulation of adaptive immunity by the innate immune system. *Science*.

[31] Manicassamy S, Pulendran B (2009). Modulation of adaptive immunity with toll-like receptors. *Seminars in Immunology*.

[32] Steinman RM, Hawiger D, Nussenzweig MC (2003). Tolerogenic dendritic cells. *Annual Review of Immunology*.

[33] Jing Y-H, Chen K-H, Yang S-H, Kuo P-C, Chen J-K (2010). Resveratrol ameliorates vasculopathy in STZ-induced diabetic rats: role of AGE-RAGE signalling. *Diabetes/Metabolism Research and Reviews*.

[34] El-Mowafy AM, White RE (1999). Resveratrol inhibits MAPK activity and nuclear translocation in coronary artery smooth muscle: reversal of endothelin-1 stimulatory effects. *FEBS Letters*.

[35] Buttari B, Profumo E, Mattei V (2005). Oxidized *β*2-glycoprotein I induces human dendritic cell maturation and promotes a T helper type 1 response. *Blood*.

[36] Profumo E, Buttari B, Riganò R (2009). Oxidized haemoglobin as antigenic target of cell-mediated immune reactions in patients with carotid atherosclerosis. *Autoimmunity Reviews*.

[37] Profumo E, Buttari B, Alessandri C (2010). Beta2-glycoprotein I is a target of t cell reactivity in patients with advanced carotid atherosclerotic plaques. *International Journal of Immunopathology and Pharmacology*.

[38] Buttari B, Profumo E, Capozzi A, Sorice M, Riganò R (2011). Oxidized human beta2-glycoprotein i: its impact on innate immune cells. *Current Molecular Medicine*.

[39] Profumo E, Buttari B, Riganò R (2011). Oxidative stress in cardiovascular inflammation: its involvement in autoimmune responses. *International Journal of Inflammation*.

[40] Yan SF, Ramasamy R, Naka Y, Schmidt AM (2003). Glycation, inflammation, and RAGE: a scaffold for the macrovascular complications of diabetes and beyond. *Circulation Research*.

[41] Nienhuis HL, de leeuw K, Bijzet J (2008). Skin autofluorescence is increased in systemic lupus erythematosus but is not reflected by elevated plasma levels of advanced glycation endproducts. *Rheumatology*.

[42] Nienhuis HLA, Westra J, Smit AJ, Limburg PC, Kallenberg CGM, Bijl M (2009). AGE and their receptor RAGE in systemic autoimmune diseases: an inflammation propagating factor contributing to accelerated atherosclerosis. *Autoimmunity*.

[43] Tan ALY, Forbes JM, Cooper ME (2007). AGE, RAGE, and ROS in diabetic nephropathy. *Seminars in Nephrology*.

[44] Wautier M-P, Chappey O, Corda S, Stern DM, Schmidt AM, Wautier J-L (2001). Activation of NADPH oxidase by AGE links oxidant stress to altered gene expression via RAGE. *American Journal of Physiology*.

